# Exploring the role of spirituality and the meaning of life of family caregivers: a qualitative study in Germany

**DOI:** 10.1186/s12912-025-03398-x

**Published:** 2025-06-27

**Authors:** Jenny Kubitza, Verena Steinmaier, Natascha Lauer, Anna Pendergrass, Eckhard Frick

**Affiliations:** 1 TUM School of Medicine and Health, Department of Psychosomatic Medicine and Psychotherapy, Spiritual Care and Psychosomatic Health, Technical University of Munich, TUM University Hospital, Munich, Germany; 2 Centre for Health Services Research in Medicine, Department of Psychiatry and Psychotherapy, Uniklinikum Erlangen, Friedrich-Alexander-Universität Erlangen-Nürnberg (FAU), Erlangen, Germany

**Keywords:** Caregiving needs, Family caregiver, Informal caregiving, Spirituality, Qualitative study

## Abstract

**Background:**

Family caregivers suffer from multiple strains, e.g., social isolation or lack of leisure time, which has a negative impact on their health, well-being, and quality of life. Spirituality may support in coping with the family caregivers’ burdens. There is currently a lack of studies examining how family caregivers experience the meaning of life and how spirituality helps them to cope. More research is needed to provide quality care to people in need of care and their family caregivers.

**Methods:**

The study has an exploratory-descriptive qualitative research design based on semi-structured interviews conducted with 24 family caregivers in Bavaria, Germany. The data generated were transcribed verbatim, and a content analysis approach was used for data reduction, obtaining analytical code, and determining categories.

**Results:**

Caring for a relative often leads to changes in the lives of family caregivers, which can affect their experience of meaning. The experience of meaning includes four dimensions: (1) values, (2) aspects before caregiving (past), (3) current situation (present), and (4) expectations after caregiving (future). The experience of meaning goes beyond the current care situation, family caregivers rather clarify what defines their life and how care becomes part of this life. If family caregivers experience meaning in their lives, it helps them to accept their care role, develop a better connection to themselves, and become generally more aware of themselves as individuals with needs and requirements. Three forms of spirituality help family caregivers to experience meaning in life: reflection, awareness, and inner confidence.

**Conclusion:**

Regardless of the illness and the specific care needs of the relative, clarifying the meaning of one’s own life is essential to accepting the role of the caregiver and coping with it positively over a long period. Many family caregivers benefit from professional support in this reflective and dynamic process.

**Clinical trial number:**

N/A.

**Supplementary Information:**

The online version contains supplementary material available at 10.1186/s12912-025-03398-x.

## Introduction

Most people with long-term needs are cared for by relatives, neighbors, friends, or community members at home [1, 2]. In Europe, almost 80% of people with care needs receive care at home through relatives, with or without additional professional care [2]. A person who provides care without payment for a family member or a friend, assisting with physical, cognitive, or emotional aspects of life on a daily or intermittent basis, is called a *family caregiver* or *informal caregiver* [3]. The concept of family caregivers (FC) will be used throughout the manuscript, as the study focused on caring for family members.

Wolff et al. surveyed 2.122 FCs in their study about the help they provide; support with household activities is most frequently provided (95.7%), followed by transportation (76.8%), and mobility (67.5%). In addition, more than half of the FCs regularly support their loved ones with self-care (50.4%), medication management (52.9%), and healthcare activities (59.1%). The FCs care for their loved ones for an average of 13.9 h a week; if the person has dementia, the effort for the FCs increases significantly (31.0 h per week) [4].

FCs often have a high level of responsibility, restrict their previous lives, and tend to neglect their own health [5, 6]. In a recent study of 27.000 FCs in Germany, two-thirds of the respondents reported suffering daily discomfort, and more than a third expressed the opinion that caregiving is difficult or even impossible to manage [6].

FCs are co-helpers and co-sufferers and should be supported according to their needs and burdens. Research is required on the needs of FCs, especially their spiritual needs. Previous studies indicate that illness and the need for care often result in an imbalance in families and lead to life-changing situations. Most FCs have to reorganize their everyday lives and change their life plans, which usually shatters their meaning in life [7, 8].

The concept of spirituality is often related to the meaning of life and connectedness. A systematic review that evaluated the most important and highly cited definitions of spirituality in the healthcare field identified 24 dimensions of spirituality and concluded that spirituality is primarily regarded as a connection or relationship that provides (or is the search for) meaning or reason for being. This connection can relate to God or a higher power, to something transcendent, to other people, through self-connection, and/or with nature. Spirituality was presented as an intrinsically human characteristic composed of beliefs or faith, experiences, and practices or behaviors. Spirituality is a dynamic process associated with the development of healing, reconciliation with self or others, peace and feelings of well-being, values, and personal growth [9]. However, this study is not the only one to recognize that spirituality is always subjective and individual and that the concept is too complex to be defined comprehensively and conclusively [9, 10]. Roser finally concluded that spirituality is precisely and exclusively what the affected person believes it to be [11].

In their reviews, Benites et al. (2021) and Casaleiro et al. (2022) systematically examined the spirituality of FCs of persons with mental illness or in palliative care situations. Their findings indicate that as a result of the illness and caring for their relative, FCs begin to reflect on their own lives, search for new life content, and thus learn to accept the new life situation [7, 8]. FCs who accept the new life situation show fewer symptoms of existential suffering, anger, helplessness, feelings of abandonment, and the inability to find meaning in life [7].

In other clinical contexts, FCs’ spiritual resources were primarily investigated. The most studies identified reflection [12–22] and acceptance [14, 15, 17–19, 21, 22] as spiritual resources for FCs. Hope and faith help the FCs continually to strengthen their optimism [13–15, 18–21, 23–25], to see caregiving as an opportunity to show their best, and be rewarded by God for it [14, 16–18, 21].

All studies mentioned the experience of meaning as part of the FCs´ spirituality, but only one literature review explicitly examined how the experience of meaning affects FCs. Meanings in stroke family caregiving were interpreted as an obligation resulting from moral, religion, expectations, and a subjective choice based on love, hope, and a sense of reciprocity. They concluded that FCs need to experience meaning in their loved one’s illness and care, then they can continue their care efforts [26].

However, there is a lack of studies investigating how FCs can determine the search for meaning for themselves despite the challenges of domestic care and which resources help them in their search for meaning. Expanding this knowledge will support the provision of spiritual care tailored to FCs. It will enhance the ability of healthcare personnel to provide appropriate, individualized spiritual care to patients and their FCs and address the desire of FCs to be supported in their existential and spiritual needs [27]. Therefore, the aim of this study is to explore how FCs experience their spirituality and search for meaning. The following research questions were formulated: How do FCs experience spirituality and search for meaning? Which spiritual resources help them experience meaning in life?

## Methods

### Study design

The qualitative study examines the spiritual needs and requirements of FCs. This study has an exploratory-descriptive qualitative (EDQ) research design [28] based on individual interviews with FCs. The study is reported in accordance with COREQ (COnsolidated criteria for REporting Qualitative research) Checklist [29]. The complete checklist can be found in the appendix (see Additional File 1).

The Centre for Health Services Research in Medicine at the University Hospital Erlangen and at the University of Erlangen collaborated on the study. Their quantitative study explored the FCs´ use, their needs, and also the effects of the usage of formal support services on various aspects of FCs’ lives.

### Sample and recruitment

For the recruitment, flyers were handed out personally to FCs at self-help groups (*n* = 125) by the first author and uploaded to social networks for FCs (*n* = 2 posts). The recruitment process also involved collaboration with the cooperationspartners of the quantitative study. The quantitative survey was supported by the Medical Service of the Bavarian Health Insurance (MD). In Germany, care assessors from the MD visited the FCs at home and assess patients’ care needs. As a part of this process, the MD distributed quantitative questionnaire for voluntary participation. FCs who participated in the quantitative survey were offered the opportunity to receive personalized information about their health risks associated with home care. Additionally, they could consent to a quantitative follow-up survey by voluntarily providing their address. Those who provided their address received, alongside the health risk information, a flyer containing details about the qualitative survey sent by mail (*n* = 404). People interested were requested to contact the research team directly by mail or telephone.

FCs were recruited for the study if they (a) were at least 18 years old, (b) were caring for a relative in a domestic setting, and (c) had sufficient knowledge of German to understand the informed consent and complete the interview. To adhere to the desired diversity of the FCs, it was planned that the participants should differ in their characteristics, e.g., age, gender, and duration of care. A total of 24 interviewed FCs were included in the study.

Table 1 reports the characteristics in detail.

**Table 1:** Demographic information on the participants (*n* = 24)

Characteristics	Female Caregivers (*n* = 14)	Male Caregivers (*n* = 10)
Age	61 years (32–77 y)	65.6 years (47–81 y)
Daily care time	7.2 h (0.5–24 h)	3.7 h (1–8.5 h)
Duration of care	6.3 years (1–30 y)	5 years (0.5–17 y)
Relational status of person in need of care	7 spouses	5 spouses
	6 parents	4 parents
	1 child	1 child
Illness of the person in need of care	5 physical	4 physical
	0 psychosomatic	1 psychosomatic
	9 cognitive	5 cognitive
Need for care according to German grading system (from one = low to five = high)	1 one	2 one
	6 two	4 two
	4 three	2 three
	2 four	2 four
	1 five	0 five
Employment	3 full-time	5 full-time
	2 part-time	0 part-time
	3 unemployment	0 unemployment
	6 pension	5 pension
Religious affiliation	3 Catholic	4 Catholic
	5 Protestant	2 Protestant
	0 Muslim	0 Muslim
	0 Jewish	0 Jewish
	1 Non-practicing religious	3 Non-practicing religious
	5 Atheist	1 Atheist

### Data collection

Data were collected between August 2022 and July 2023. Semi-structured interviews with FCs in Bavaria, Germany, were conducted face-to-face, via telephone, or video conference - as preferred by participants. The interviews ranged from 27 to 90 min with an average time of 45.5 min, with interviews with men taking slightly longer (ø 51.6 min) than with women (∅ 41.15 min). The interviews were conducted by the first author and two student assistants.

A total of 44 people agreed to participate in the study. After 21 interviews, no new themes were identified during the analysis [30]; three additional interviews were conducted to confirm the developed categorization system and ensure data saturation was reached.

A semi-structured interview guide was used. The interview guide was developed by two authors (EF, JK) in five phases: (1) identifying the prerequisites for using semi-structured interviews; (2) retrieving and using previous knowledge; (3) formulating the preliminary semi-structured interview guide; (4) pilot testing the guide; and (5) presenting the complete semi-structured interview guide [31]. During the interviews, the interviewer encouraged the participants to describe their experiences in care. To ensure that the whole spectrum of experience was covered, the participants were asked about different aspects of care and how these aspects related to their spirituality. The guideline was flexibly adapted to the interview situation (see Table 2). The first two interviews were used to test the interview guide. As the interviews resulted in relevant data, they were included in the analysis.

**Table 2:** Theme, stimuli, and guiding questions of the interview guide

Theme	Stimuli	Guiding questions
Experiences	First of all, I would like to find out how you care for your relative on a typical day.	What kind of care do you provide? How has the meaning of your life changed since you began caring for your relative? Do you feel that caring for your relative affects your inner peace/spiritual feelings? Are you aware of your spirituality?
Benefits Meaning	I would like to know about any positive experiences you had since you started caring for your relative. Please tell me what caring for your relative means to you.	How would you describe your physical, psychological, social, and spiritual well-being? What does spiritual well-being mean to you? What motivated you to take on the role of caregiver? How have you interpreted your relative’s need for care? How has your relative’s need for care changed your beliefs?
Challenges and burdens	Please tell me about situations that you experience as particularly difficult while caring for your relative.	What aspects of care are particularly difficult for you? How have you responded to these difficulties and what strategies have you used to cope with the burdens? Have you discovered new ways to express your spirituality?

### Data analysis

All interviews were digitally recorded and transcribed verbatim by the authors. The first three transcripts were checked for accuracy by at least two researchers by comparing the transcripts with the audio files. This resulted in a total of 469 pages of interview transcripts. The transcripts were imported into MAXQDA version 22.7.0 and analyzed inductively [32]. The inductive analysis aimed to reduce the amount of text so that the essential content was retained. Therefore, the material was abstracted in several steps to create a category system [28, 32] (see Table 3). The first and second authors mainly conducted the data analysis. The results were presented to the rest of the research team in two qualitative workshops and discussed until all agreed.

**Table 3:** Steps of content analysis with examples

Steps	Researcher activity	Examples
1	Multiple readings of transcripts, identification of relevant text components that answer the research question (JK, VS)	*“Whenever I hear something like that [someone needs help]*,* I just jump right in and take care of it; and now I’m just about to stop doing that.“*
2	Paraphrasing of identified text components (JK)	FC wants to stop always helping everyone.
3	Identification of paraphrases with similar content to generate codes (JK, VS)	FC wants to find a new conduct for situations in which she is asked for help. FC is reflecting her previous behavior of always wanting to help everyone.
4	First data reduction (deleting similar paraphrases within one interview and non-essential paraphrases) (JK)	FCs see care as opportunity to change old behaviors and develop themselves further.
5	Second data reduction (bundling similar paraphrases into categories) creating category system (JK, VS)	*Development* as a category
6	Review of the category system to ensure that all originally formulated paraphrases were included in the new system (five team members of the professorship for Spiritual Care and Psychosomatic Health)	*Development* as a subcategory of *reflection*
7	Review of summarizing category system using the source material (JK, VS)	*Development* as a subcategory of *reflection* wih 40 codes in 16 transcripts

### Ethics

The Technical University of Munich Ethics Commission reviewed and approved this study (Project ID: 2022-416-S-ND). All participants received verbal and written information about the study. To prevent the FCs from feeling pressured into participating in the study and to take their vulnerability into account, the researchers provided a clear and transparent informed consent process. They informed the participants that their responses or participation would not affect their caregiving role or relationship with any involved institutions or caregivers. Moreover, before interviewing, the researcher reiterated that participation was entirely voluntary and that choosing not to participate would not affect any services they were receiving. Interviews were conducted with sensitivity to topics that could trigger distress, and breaks were offered if necessary. The researchers were independent persons who had no influence on their care situation. This might help ensure that FCs were not exploited or unduly influenced. The participants did not receive any benefits for their participation. None of the participants invited withdrew from the study.

Written informed consent was obtained from all participants in paper form and stored in the hospital offices, which were only accessible to authorized persons. They were able to withdraw their consent until the data were analyzed. The participants´ personal and sensitive data were anonymized during transcription. The personal data and the written informed consent were deleted after the study ended. The transcripts will be deleted ten years after the end of the study [33].

### Quality, rigor, and trustworthiness

Strategies to ensure trustworthiness were parts of the qualitative methods in this study and were considered in the (a) preparation, (b) organization, and (c) reporting phase [34].

- Preparation phase: Semi-structured interviews are a suitable method (1) to collect the personal and individual experiences of spirituality among FCs and (2) to address specific relevant topics. As each topic began with a stimulus, the FCs gave the information that was relevant to them. The interview guide was pre-tested with FCs, but could also be flexibly adapted during the interviews. The interviews were conducted by two student assistants and one researcher who is experienced in interviewing. The researcher coached the two student assistants before their first interviews. This involved practicing various interview techniques in the context of short simulated interviews, e.g. active listening, paraphrasing, or asking questions. In the further process, all interviewers regularly gave each other feedback about the others’ interview skills and discussed whether the interview guide needed to be adapted. The first and second authors mainly conducted the data analysis, who discussed which analysis units should be considered together.
- Organization phase: All researchers are familiar with qualitative content analysis. During the analysis, the main categories and subcategories were discussed several times between the first and second author, and the category system developed was reviewed twice in qualitative workshops until a discursive agreement was reached between all researchers (five team members of the professorship for Spiritual Care and Psychosomatic Health).
- Reporting phase: The results were presented according to the category system developed. Correlations between the (sub)categories were explained. Quotes from the interviews supported all categories.

## Results

Most FCs assisted with personal hygiene in the morning and evening and prepared meals and medication three times a day. FCs of people with cognitive impairment or dementia supervised their family members during the (entire) day and felt responsible for their activities. FCs of people with severe physical disabilities regularly mobilized those affected and assisted them with their eliminations, sometimes several times a night. FCs of people in need of less care mainly managed the housework for the affected person, e.g., grocery shopping or tidying up the house. Almost all FCs were responsible for bureaucratic and administrative activities for the person in need of care.

Table 4 prestens the final category systems, organized in two main domains ‘The meaning of life’ and ‘Spiritual resources’. The first main domain includes four categories (values, past, present, future) and the second main domain includes three categories (reflection, awareness, inner confindence).

### The meaning of life

Regardless of the period during which FCs provided care, both groups of FCs who had been caring for a short or very long time struggled to find meaning in the care situation and their lives. Two participants stated:

I have no meaning in life anymore. The sense of my life consists of caring for my husband. I have no private life anymore.

(Wife, 67 years old, 31 years of care)

As long as I can still go to work and don’t have to give up my whole life; I wouldn’t do that now; that I’m only there for the sick person.

(Mother, 56 years old, 13 years of care)

The findings indicate that FCs who predominantly identified as caregivers rather than individuals with their own needs rarely experienced meaning in life, mainly spoke negatively about the care situation, or struggled with it. FCs who found meaning in life were generally more aware of themselves as a person with their own needs and were more capable of separating themselves from caregiving.

To experience meaning in their lives, FCs primarily dealt with four aspects: values, past, present, and future.

### Values

FCs often explained their behavior in life with their value system. They distinguish between their own beliefs and the expectations from others. It is not uncommon for FCs to identify themselves as hardworking, disciplined, and responsible.

but that’s also because you’ve been disciplined your whole life (…) and (…) I’m very aware that I have a responsibility.

(Wife, 68 years old, 2.5 years of care)

These are values that they have held all their lives. In the caregiving process, they often realize that some values have been imposed on them from others. One FC stated that it is important to her that *“everybody is happy and satisfied”* (Daughter-in-law, 32 years old, 1 year of care). This is a good example of a value that could hardly be realized anymore due to home care or only with relevant burdens, which often initiated a rethink among the FCs. The FCs reflected on their beliefs to differentiate which values are important to them and which expectations others impose on them. One of the participants expressed:

At most, you castigate yourself even more. There is (…) a passage [in the Bible] that tells us (…) that children should take care of their parents.

(Son, 47 years old, 2 years of care)

FCs often only realize through the caregiving that proven values came from others and exerted pressure. Caregiving enabled them reflect on their value system which helped them to experience meaning in care.

### The past

FCs often used their past experiences to explain why they took on the role of caregiver and experienced meaning in caregiving. This also included their biography, in particular, previous experiences with care, and the death of a loved one; for example, one wife explained that her father died alone in a hospital, so she promised herself at this moment that all her family members would die at home from then on:

My father died alone in hospital. (…) and this triggered such a trauma in me that I unconsciously said, please let my relatives die at home.

(Wife, 67 years old, 30 years of care)

The past also included numerous positive experiences with the person requiring care. Almost all FCs were motivated in their care provision to give something back to the person they care for. This was particularly apparent in FCs who care for people with cognitive impairments.

He did a lot for us children in his life. He was always there for us. (…) and I thought to myself, now I can also be there for him and do for him what he needs.

(Daughter, 60 years old, 3 years of care)

Some FCs reported that it means a lot to them that their relative receives the best possible care. Based on their mostly negative experiences with professional care, they decided they would rather provide the care themselves and thus ensure good care.

For my wife, it’s quite different from being in a residential home. I was a volunteer carer for two people in a residential home. Depending on the situation, they sit there for hours and are completely apathetic at the table. (…) of course, it’s completely different when my wife has constant contact and assistance with us at home.

(Husband, 73 years old, 11 years of care)

### The present

At present, it became apparent that FCs perceived the challenges and restraints caused by caregiving. To experience a sense of purpose in care despite burdens, several FCs consciously focussed on the benefits caused by caregiving. The benefits predominantly refered to social aspects, not only relating to the person requiring care. FCs also mentioned that the solidarity within their family or the support from friends and self-help groups became stronger, as one of the participants described:

So I think that it also strengthens a family, right? (…) I think the [relationship with my brother] has also become stronger because we talk to each other more. We have to discuss more things with each other.

(Son, 53 years old, 1 year of care)

Another benefit is that some FCs also learnt to have compassion for themselves. This helped them to consciously maintain boundaries in their care- an important strategy of self-protection. The following quotes illustrate how one FC exerts boundaries, another does not:

I’m disgusted [by intimate hygiene]; (…) He’s my husband, and we had a sex life before (…) I wouldn’t do that. (…) I would give my husband to a professional.

(Wife, 75 years old, care for 11 years)

My [own needs] are gone. Absolutely gone. I go to work. Do the housework (…). In between, I’m just there for the wife. That’s it.

(Husband, 58 years old, care for 17 years)

### The future

FCs often explained caregiving by imagining their future with the person in need of care. FCs described various wishes for the future; some FCs wanted to preserve the health of the person in need of care and spend as much time as possible with them, while other FCs wished to accompany the person in need of care to a pleasant death. These wishes gave the FCs a perspective and meaning in life.

We both firmly believe (.) that there is such a thing as rebirth (.) for me personally (…) that makes an amazing amount of sense.

(Husband, 72 years old, care for 10 years)

It also helped if the FCs already had expectations for future aftercare, as they could experience a sense of purpose beyond care.

Now I’m retired and now I want to travel while I’m still relatively healthy (…) and at the moment I’m just very, very limited with this. and (…) from a rational point of view, [the limitation] will only end when my father dies.

(Daughter, 66 years old, 3 years of care)

### The spiritual resources

Every participant mentioned aspects that can be seen as spiritual resources essential to regain strength and experience meaning in life. One husband impressively described the strengthening he experiences through his type of spirituality:

When I go out, I’m (…) with myself and when I look at the landscape, (…) where I know that the trees used to be small and are now enormous. (…) That makes me happy.

(Husband, 81 years old, 4 years of care)

A daughter said:

Every day, at least two hours of prayer, meditation, something like that, and that’s my source of strength. (…) That’s where you get distance from things; (…) I’ll admit, the person who doesn’t use this has to go out; they have to go for a walk, (…) to get distance from things; and through meditation, you don’t have to leave your flat, and still get the total distance.

(Daughter, 60 years old, 0.5 years of care)

The data analysis revealed that FCs mentioned three forms of spirituality that help them experience meaning in life: reflection, awareness, and inner confidence.

### Reflection

FCs explained that the person’s illness confronted them with their vulnerability, and therefore, they critically examined their own lives. They consciously questioned what was meaningful in their lives and what they should let go of. Two participants shared their reflections:

It can also be over very quickly. It was already the case that I (…) was even more confronted with my mortality.

(Daughter, 52 years old, 2 years of care)

Whenever a person leaves, you are spiritually challenged (…), and I think dealing with it helps me progress spiritually. In the sense that I am constantly reassessing where I am at the moment.

(Son, 58 years old, 3 years of care)

Several participants agreed that reflecting on their lives contributed to their development. Almost all FCs said that they experienced personal development. One woman reported that they know her needs now and can set boundaries:

One issue for me is always saying no. But of course, these are things (…) I have to find my way, (…) but I can now say no to … (name of the person in need of care).

(Wife, 53 years old, 1 year of care)

Reflection also made it easier for FCs to accept the illness of their relative and the caregiving as part of their lives. They concluded that caring is an assignment they have been given to accomplish. Accepting the situation sometimes required a longer reflective process; in particular, FCs who blamed the person for requiring care struggle with the care situation, as two of the participants stated:

For me, it feels absolutely right now. That’s just what life wants from me now.

(Daughter-in-law, 51 years old, 10 years of care)

But you’re sad when something like that happens, right? But on the other hand, (…) it’s also her fault. (…) When it snows or rains and then these shoes that she had on when it happened. It didn’t have to happen, right? And that’s where I struggle afterwards. (…) It freaks you out. (…) It’s always easy for her. (…) What happened happened; I can’t change it. Yes, of course not, but it shouldn’t have happened (…) I often scold her.

(Father, 79 years old, 2 years of care)

### Awareness

The confrontation with the person’s illness requiring care also led to the FCs becoming more aware of the positive aspects of their lives. The previous daily life was often more appreciated, for example, being thankful for their health, having friends, being financially stable, or having hobbies.

I’m more interested in health now; maybe I’ve learned that for life. The other thing for me is (…) I think you have to deal with situations in a more relaxed way; somehow, you find a solution.

(Son, 53 years old, 1 year of care)

Creative activities such as art, music, or books helped FCs to consciously focus on the moment and to connect with themselves:

My thoughts are always with my mother. (…) I’m only with myself when I can immerse myself in a book.

(Daughter, 62 years old, 4 years of care)

### Inner confidence

Religious FCs often mentioned faith as a spiritual resource. Faith helped them accept the situation as God’s assignment, prayer allowed FCs to centre and contemplate, and the church community was perceived as a great support.

At least two hours of praying every day (…) and that’s the source of strength; without that, there is no chance. (…) It’s also where you get distance from things; so (…) the person who doesn’t use this tool, they just say (…) I have to get out.

(Daughter-in-law, 51 years old, 10 years of care)

Hope enabled FCs with and without a religious background to introspect and advocate. Religious and non-religious people hoped (a) that the condition of the person in need of care would remain stable or improve, (b) that they would be able to cope with everyday life, and (c) that one day they would receive the good they had been given in return.

I always try to adopt a calm attitude, because at some point it might be me who needs care, and I (.) want my children to learn (…) that’s what you do.

(Son, 47 years old, 2 years of care)

**Table 4:** Category system of the qualitative study

Main categories	Categories	Subcategories	Description	Quotes
Meaning of life	The values	Own beliefs	If FCs can fulfil their own beliefs through care (e.g., responsibility or helpfulness), they experience a sense of meaning in it.	*“Yes*,* it’s kind of a matter of course*,* isn’t it? (…) that takes time*,* of course*,* (…) and at the same time (…) so if a family doesn’t stick together in a situation like that*,* then you don’t really need one. so that’s how we thought about it.* (Son, 53 years old, 3.5 years of care)
		External expectations	FCs realized through caregiving that some of their values were more a result of the expectations of others (e.g., not refusing requests). This realization reduced pressure in several areas of their lives, which caused them to recognize an important meaning in their caring activities.	*“I always wanted to be the lovely child. (…) I just realized if you’re nice (…) then it’s a bit easier in life*,* and that’s how this role developed in me*,* (…) always looking how others are feeling.”* (Daughter, 60 years old, 3 years of care)
	The past	The biography	If FCs had terrible experiences with caring for or losing a loved one, it means much to them to make it better this time.	*“My father was a very heavy care case for four years and we couldn’t manage it at home*,* (…) and then he went into a nursing home*,* and we went there every day. (…) I can’t go in there and I can’t give my mum in there either*,* and my mum would die if she had to go into a nursing home. That’s why I care for her*.“ (Daughter, 62 years old, 3 years of care)
		The story with the person in need of care	FCs want to give the family member the same things back through caregiving that their family member has given them for years (e.g., love, attention). These thoughts mean a lot to the FCs and give them energy for caring.	*“You try to give everything for that person because he deserves it. He was always there for us. He always did everything for us. (…) I’m pleased that I can help.”* (Wife, 75 years old, 2 years of care)
		The story with the care	If FCs had negative experiences with professional care, they prefer to care for their relative themselves because it means a lot to them that their family member receives the best possible care.	*“I had my husband in a residential care home for four months. (…) My husband was (…) desperate. That wasn’t good for me either*,* because I saw that his care needs weren´t met and that he was suffering*.“ (Wife, 67 years old, 30 years of care)
	The present	Burdens	The burdens hurt the FCs’ experience of meaning through care (e.g., distance from the person in need of care, isolation, self-doubt).	*“I don’t think you can talk about things the way you used to (…) you can tell her something*,* but she doesn’t know it anymore. (…) There’s nothing left (of her).”* (Husband, 81 years old, 3 years of care)
		Benefits	If the FCs recognize that they experience benefits through care, they attribute meaning to it (e.g., strengthening of family solidarity, growth of character).	*“I would like to say that our relationship has also improved; I can tell that my husband is very grateful for the loving and very good care I give him.“* (Wife, 77 years old, 10 years of care)
	The future	Certainty	FCs are aware that there will be a time after care and are already visualising it. These visions give them the strength to cope with the current situation.	*“and in the end (the death of my mother) will also be a relief for me. (…) So I can (…) look forward to it in a positive way and say that’s a good thing. (…) Then I will have a bit more freedom again. Time for myself.“* (Daugther, 52 years old, 1 year of care)
		Desires	If the FCs have wishes regarding the person in need of care and they can contribute to their fulfilment, they experience meaning in their care work (e.g., a peaceful death).	*“I want him to die at home. (…) how beautifully my friend organized it. The grandchildren played in the living room where the man was lying. The children were there; they talked. The palliative doctor was there; he gave him an infusion so that he was no longer in pain. The pastor was there. They drank beer together. And they talked - and very slowly*,* this man went into the afterlife. And this is my dream of how I would like it to be*.” (Wife, 67 years old, 30 years of care)
Spiritual resources	Reflection	Acceptance	FCs who accept the person’s illness and care experience inner peace.	*“This sadness that accompanied me for years. It was like a dark cloud that I couldn’t shake off. It was just always there (…) but it’s gone now. (…) The situation hasn’t changed*,* but I’m fine again.*“ (Wife, 77 years old, 10 years of care)
		Development	FCs who recognize that they have developed as a result of care accept of care.	*“My father takes a lot of things I do for granted; I’m sometimes a little disappointed*,* and he (the supervisor) encouraged me to (…) [demand] a thanks. (…) I’m always working on that and always learning and always reflecting*.“ (Daughter, 60 years old, 3 years of care)
	Awareness	Appreciation	FCs appreciate the contents of life more, perceive them more consciously, and in some cases develop a sense of awe, e.g., health, time, nature.	“*A different attitude to the value of time. (…) A care relationship like this creates a very sensitive awareness of time. For the time that you have for yourself.”* (Son, 58 years old, 3 years of care)
		Creativity	FCs consciously take time to fulfil their creativity. In these moments, they forget about the care and allow their minds to fully immerse themselves in the moment.	*“My business card says local history researcher and that’s what I enjoy doing. I’ve now published (…) 8 books on local history. (…) I draw an incredible amount of energy from it.*“ (Husband, 73 years old, 10 years of care)
	Inner confidence	Faith and religious practices	Faithful FCs gain confidence and strength through religious practices such as prayer or bible reading. The members of the church in particular are a powerful source of strength.	*“We are very religious and of course it helped us a lot (.) that many people prayed for us*.“ (Mother, 56 years old, 12 years of care)
		Hope	Hope gives FCs confidence and strength. Hope relates both to the present (e.g., the condition of the person in need of care remains stable) and to the future (e.g., I will receive back what I have given).	“*At worst*,* he’ll have to go into a residential home*,* but I hope that this happens very late. I’m there for him (…) and I’m trying to manage it as well as possible at the moment*,* so that both of us have an acceptable quality of life*” (Wife, 75 years old, 5 years of care)

## Discussion

1. The FCs in this study reported struggling to experience meaning in the care situation and their lives. In this study, it became apparent that the experience of meaning was initially shaken. However, most FCs tried to experience meaning and clarity as to why their family member’s illness and care had entered into their lives. If FCs could not address the existential aspects, they did not appear to be in harmony with the situation. They were barely able to reconcile the illness and the associated limitations with the family member. Many FCs said that they no longer recognized their family member. One FC even seemed angry with the person in need of care.

Haußmann et al. identified similar findings: caring for a loved one at home changes one´s life radically; FCs adapt their priorities and routines, reorganize their daily routine, and adjust their previous life plans, which initially shatters the meaning of most FCs’ lives [35]. The experience of meaning is a core element of spirituality that can energize and help to cope with crises [9]. Sena et al. highlight that spirituality is a dynamic process that helps to develop feelings of healing, reconciliation with oneself or others, and peace in crises [9]. According to Puchalski & Ferell, feelings of healing and reconciliation with oneself or others can only arise when meaning can be experienced in the crisis [36]. Therefore, it appears that the experience of meaning is essential for FCs to reconcile with and accept the care situation. Zhang & Lee even assume that the experience of meaning is necessary for FCs to continue their care efforts [26].

The study indicates that FCs can experience the search for meaning on different levels. Meaning is not always experienced only in the present; other levels, such as the past and the future, as well as values, influence the experience of meaning. This becomes especially apparent in the example of religious FCs. In this study, religious FCs used their faith (value) to experience meaning. They used their faith to motivate them to care for their family member: they spoke of love for others or that it was a task given to them by God, similar to previous studies [14, 16–19, 21]. However, this study also highlighted that faith could become a burden if FCs feel they cannot meet the high demands of caregiving, which hurts their sense of meaning. The study by Haußmann also explored this ambivalence; highly religious FCs can benefit more from faith as a resource than less religious FCs, but they are also more affected by internal conflicts [35]. Nemati et al. observed in their qualitative study that FCs may perceive it as a punishment from God and consider it unjust [18]. In the study by Opsomer et al., religious FCs believe that they and their relatives don’t deserve this [19].

This differentiation of levels complements previous studies, which tended to use the wording entire life situation to describe how FCs can reflect on the care situation to experience meaning [7, 8], but also illustrates the complexity of the experience of meaning. That study also confirms that FCs cannot always experience meaning due to the burdens and negative emotions, regardless of the length of time they have been caring for a family member [7, 8, 37]. According to Danucalov et al., this is often because FCs are forced to be more externally orientated due to the care they provide and only perceive themselves to a limited extent [37].

2. Three spiritual resources emerged as valuable for FCs to experience meaning: (a) reflection, (b) awareness, and (c) inner confidence.

- Reflection can lead to acceptance and development. This study further identified that spirituality helps people reflect on their development. Although personal growth and knowledge of oneself are usually triggered by caregiving, this development is not solely dependent on care. It can be seen as a benefit that extends beyond caregiving. These benefits help FCs to experience meaning in caregiving and to embrace the situation.

In previous studies, reflection and acceptance were identified as the most common spiritual resources that help FCs become aware of their own needs and develop a mindful relationship with themselves [7, 8, 12–22].

b) Awareness can be expressed through appreciation and creativity. FCs were particularly grateful for their health, but other aspects, such as social networks, hobbies, and finances, were also mentioned.

Previous studies have noted that FCs value their health more and even adopt a live-for-today philosophy [16, 18, 19, 21]. This helps them to strengthen inner peace [14, 15, 17–19, 21, 22].

The FCs in this study often used creativity to strengthen awareness. They listed, e.g., painting, writing books, or singing. Sena et al. already recognized that people use art to feel a connection and experience meaning, whereby art was recorded significantly less frequently in studies on spirituality compared to Divine, God, or Higher Power (1.80% vs. 39.75%) [9].

A possible reason for the discrepancy between this study and the study by Sena et al. may be that several FCs were interviewed who described themselves as non-religious. As they hardly feel connected to Divine, God, or a Higher Power, they use new forms of connection, e.g., in the form of art. The positive effect of music and art on FCs has already been examined in further studies; a meta-analysis and a systematic review reported that art therapies significantly ameliorated caregiver burden [38] and that choral singing and visual arts interventions positively affect psychosocial outcomes [39]. However, spirituality has not yet been considered as a factor in the studies. With the rise of secular societies, it seems necessary to study music and art in relation to spirituality.

c) Inner confidence includes faith and hope. Religious FCs listed faith as one of the most important resources. FCs not only draw strength from religious practices themselves, but the religious community is also an essential resource.

In other studies, FCs already use religious practices such as prayers, reading the bible, or going to church [16, 18, 22–25]; however, other practices such as making a pilgrimage or performing ablution were also mentioned in some studies [18, 24]. FCs felt closer to God as a result, which positively affected their inner peace [16, 18, 22–25].

Our study revealed that another way to verbalize inner confidence is hope. Hope is listed as an important resource by almost all FCs in this study, regardless of their religious affiliation. It must be critically reflected that some FCs tend to hope that the situation will improve again and spend energy, time, and money on it. Although hope is an important spiritual resource, accepting that the health of the person in need of care might no longer improve at a certain point can be helpful. Other FCs see the illness of the family member as the natural course of life and express hope in different ways, such as hope that the condition can be maintained, hope that the person in need of care will die peacefully, or hope that they will still be able to spend time together.

Other studies on spirituality among FCs have already highlighted the relevance of hope [13–16, 18–21, 23–25]. In this context, a distinction between concrete and absolute hope is required. While concrete hope often occurs at the beginning of the crisis and refers to a positive and curative effect, hope changes over time to absolute hope. Absolute hope is not solution-focused but implies aspects of faith and trust that extend beyond death and external circumstances [40, 41].

**Fig. 1:**
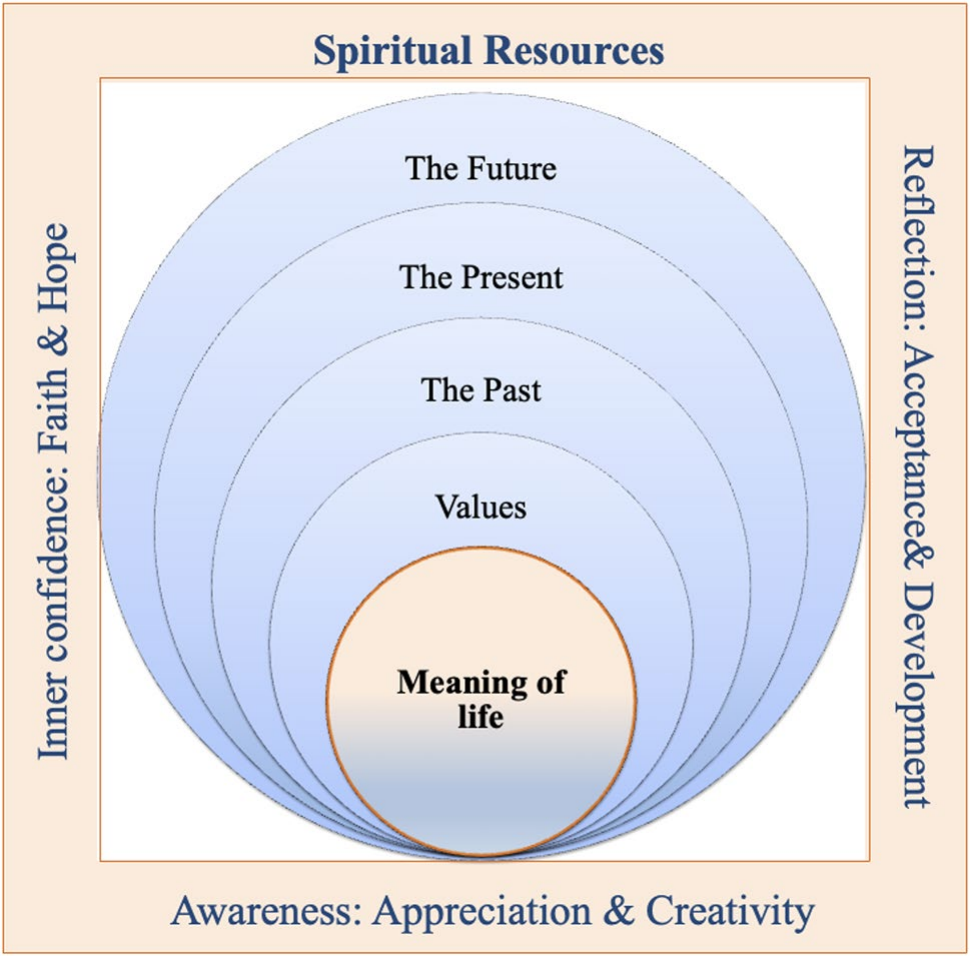
Spirituality among family caregivers

Figure 1 summarizes both research questions and illustrates how the two are connected. It represents a framework for Spiritual Resources, showing how they support inner well-being through a layered approach to self-awareness, reflection, and development. Each layer builds upon the other, guiding FCs toward greater meaning and personal growth. Figure 1 shows that spiritual resources are multi-dimensional and interdependent. Spirituality begins with a sense of self, which radiates outward through values and temporal dimensions (past, present, and future). By engaging with these layers, FCs can find strength, meaning, and resilience supported by faith, hope, reflection, and creativity.

Another result of this study is that caregiving should only be one part of the meaning of life. Although Baronet [42] and Beach et al. [43] stated that significant benefits for FCs are that they feel needed and useful through caregiving and that this positively affects the self-esteem of FCs, findings from the current study show that the feeling of being needed is to be viewed critically, as self-esteem should not only depend on caregiving. This can increase the risk of identifying oneself only as a caregiver and neglecting one’s own needs. It is also questionable what happens if the person in need of care has to move into a retirement home or dies, and informal care is no longer required.

In this study, all FCs provided examples of their spirituality, although it is apparent that they interpret the concept of spirituality differently. Some FCs dealt intensively with spiritual themes and used terms such as mindfulness, meditation, or meaning. Other FCs rarely engaged with spirituality and tended to use expressions such as connection with nature, hope, or creative work.

Viftrup et al. noted in their qualitative study that patients with a terminal diagnosis were more likely to communicate topics such as death and meaning in life with medical rather than existential vernacular. As the patients rarely spoke about their religiosity and spirituality before the diagnosis, they usually referred to the most trained vernacular with which they were familiar [40]. As the FCs in this study also rarely used the existential vernacular, some (sub)categories emerged that only partially correspond to the existential vernacular. The content analysis remains close to the material, so (sub)categories were developed based more closely on psychological-medical wording, e.g., awareness of the positive aspects of life, which is familiar to positive psychology.

Viftrup et al. recommend that patients first develop a relationship with such topics and that the dialogue partner familiarize them with the existential vernacular [40]. In future studies, FCs should first develop a relationship with the themes and clarify what the interviewee and the researchers understand by spirituality to develop a common vocabulary.

Nevertheless, spirituality is a broad and complex concept that differs according to culture, religion, and academic background and is ultimately individual. It, therefore, remains a challenge to represent spirituality holistically [9–11].

It is important to offer FCs professional support to explore their spiritual needs and activate their inner resources. Given their limited time [6] and tendency to neglect their health [5, 6], a low-threshold service can provide a valuable opportunity for FCs to reflect on their needs and burdens from an existential perspective, addressing the deeper aspects of being human.

### Strengths and limitations

24 FCs were interviewed for the study. Although the number of participants is not small for a qualitative study, it is hardly possible to collect enough data to recognize the individuality of spirituality. It is questionable whether a common understanding of spirituality can be achieved.

The data were collected using semi-structured interviews to recognize the individuality of spirituality. The triangulation of this data with a group discussion of FCs might have helped the FCs develop a common understanding of spirituality, which would have strengthened the credibility of the data.

Before the interview, the FCs were contacted several times, e.g., meetings were arranged to get to know each other. This may have made the FCs trust the researchers more, which positively affected the interviews. Nevertheless, some interviews were conducted by telephone following the participants’ requests. This may have negatively influenced the interview atmosphere, which is also reflected in the fact that the telephone interviews were shorter, with an average of 36 min, compared to face-to-face interviews (∅ 47 min) and video conferences (∅ 62 min).

The results were discussed in two qualitative workshops with researchers from various disciplines, meaning that different perspectives were considered in the analysis (medicine, nursing science, philosophy, psychology, social work, sociology). However, researchers with different clinical and research backgrounds may analyze the data differently, potentially leading to alternative interpretations or conclusions.

In case of uncertainty during data collection and analysis, a second researcher was consulted for clarification (EF, VS). This may be seen as a strength in the data analysis process.

Unfortunately, we could not conduct member checking as the results have not yet been reported back to the participants, as the study is a longitudinal study, and the evaluation of the second data collection is still ongoing.

All interviews were conducted with FCs in Bavaria, Germany. Both religious and non-religious people were interviewed, but as Germany is highly secular, the results are only transferable to other countries and cultures to a limited extent.

As the interviews were conducted in German, the quotes were translated into English. The translations were prepared by the first and second author and checked by a native speaker. However, some local words or phrases are difficult to understand in English, so regional nuances or emotional subtexts may be lost and a linguistic bias should be considered.

## Conclusions

The illness and care of a relative often lead FCs to reflect critically on their own lives. Spirituality provides a framework for understanding life as a whole in this process. Furthermore, it allows FCs to experience meaning in past and present and harmonize them with the future. It is about perceiving oneself as an individual and understanding how the provision of care can be integrated into one’s life.

FCs could greatly benefit from professional support, which encourages reflection on their entire life, including spirituality as a valuable resource. Home care and community health nurses with spiritual competencies can support FCs and play a key role in helping FCs identify and utilize their resources and address their needs early in the caregiving journey. Currently, there are limited interventions aimed at promoting spiritual coping for FCs. Developing and testing such interventions should be a priority for future research.

## Electronic supplementary material

Below is the link to the electronic supplementary material.

Supplementary Material 1

## Data Availability

The datasets generated and/or analyzed during the current study are not publicly available, due to the necessity of ensuring participant confidentiality policies and the laws of the country. However, they are available from the corresponding author on reasonable request.

## Abbreviations

FCs: Family caregivers
